# SpotCard: an optical mark recognition tool to improve field data collection speed and accuracy

**DOI:** 10.1186/s13007-019-0403-2

**Published:** 2019-02-22

**Authors:** Hamish A. Symington, Beverley J. Glover

**Affiliations:** Department of Plant Sciences, University of Cambridge, Downing Street, Cambridge, CB2 3EA UK

**Keywords:** Image analysis, Automated analysis, Field data collection, ImageJ, Fiji

## Abstract

**Background:**

When taking photographs of plants in the field, it is often necessary to record additional information such as sample number, biological replicate number and subspecies. Manual methods of recording such information are slow, often involve laborious transcription from hand-written notes or the need to have a laptop or tablet on site, and present a risk by separating written data capture from image capture. Existing tools for field data capture focus on recording information rather than capturing pictures of plants.

**Results:**

We present SpotCard, a tool comprising two macros. The first can be used to create a template for small, reusable cards for use when photographing plants. Information can be encoded on these cards in a human- and machine-readable form, allowing the user to swiftly make annotations before taking the photograph. The second part of the tool automatically reads the annotations from the image and tabulates them in a CSV file, along with picture date, time and GPS coordinates. The SpotCard also provides a convenient scale bar and coordinate location within the image for the flower itself, enabling automated measurement of floral traits such as area and perimeter.

**Conclusions:**

This tool is shown to read annotations with a high degree of accuracy and at a speed greatly faster than manual transcription. It includes the ability to read the date and time of the photograph, as well as GPS location. It is an open-source ImageJ/Fiji macro and is available online. Its use requires no knowledge of the ImageJ macro coding language, and it is therefore well suited to all researchers taking pictures in the field.

**Electronic supplementary material:**

The online version of this article (10.1186/s13007-019-0403-2) contains supplementary material, which is available to authorized users.

## Background

In many experiments, it is necessary to take photographs of biological samples. These photographs are often used for subsequent trait analysis which would be time-consuming to perform in the field, such as flower number in inflorescences or size and perimeter of flowers in pollinator experiments. It is also often necessary to record further information about the specimen in question, such as sample number, biological replicate or cultivar. Traditionally, this information has been recorded either by writing it on a piece of paper to be included in the photograph, or by making notes in a notebook or on a laptop or tablet and correlating them to the image filename. Such recording is time-consuming when in the field, requiring switching from a camera to pen and paper or a device, and requires subsequent laborious and error-prone manual transcription. Separating data capture from image capture generates the risk of mismatched data at a later stage. Various software packages exist for collecting field data (see Field Book [1], PhenoBook [2] or The Phenotyper [3] as three examples) but these are usually geared towards replicating the functionality of a notebook in a way that makes later processing less time-consuming.

Software to automate image analysis is widespread, and numerous scripts exist to measure a diverse range of plant characteristics (for examples, see [4–6]). However, such tools can only read and measure plant characteristics, and cannot extract information about the plant’s genotype, growing conditions etc.; if such information is required, it must be manually correlated to plant characteristic data at a later date.

The motivation behind SpotCard, the tool presented here, is to inextricably link plant and information within the same image in a way that reduces labour and transcription errors. ‘Mark Sense’ (also known as ‘Optical Mark Recognition’), the technique behind SpotCard, has been in use since 1937, when IBM introduced the IBM 805 Test Scoring Machine [7]. This machine detected pencil marks on paper; similar systems are widely in use today to assist in processing multiple-choice tests.

SpotCard is easy to use, has a potentially wide application area, and requires no learning of new field notebook software, design, or scripting languages. The card itself provides a convenient scale bar and a coordinate location within the image for the flower itself, and measurement of petal area or perimeter is incorporated in the macro. This allows for a one-pass process of information extraction and measurement.

Producing, using and processing SpotCard requires no specialist equipment, needing only a computer, printer, tape and glue to produce, a camera and dry-wipe pen in the field and a computer for later processing. If the camera features GPS capability (widespread in modern cameras, including Apple iPhone since 2008 and Samsung Galaxy since 2010), location information is embedded into the picture and can be read as part of the SpotCard processing. Such GPS information is generally accurate to approximately 5 m [8]. Spotcard therefore integrates notebook, camera and GPS unit into one easy-to-use tool, speeding up both data capture and data processing.

Although developed to aid in measuring flower diameter and perimeter, we see no reason why this tool could not be used when photographing whole plants, portions of plants or slow-moving animals, or in any other situation where several similar annotated images are required.

## Implementation

Spotcard is implemented as a pair of macros in Fiji [9]. Fiji is one of the leading open-source image analysis tools, being a distribution of ImageJ [10], the widely used image analysis software. It is simple to install a macro in Fiji, and users can therefore extend its core functionality.

### Producing the SpotCard

**Fig. 1:**
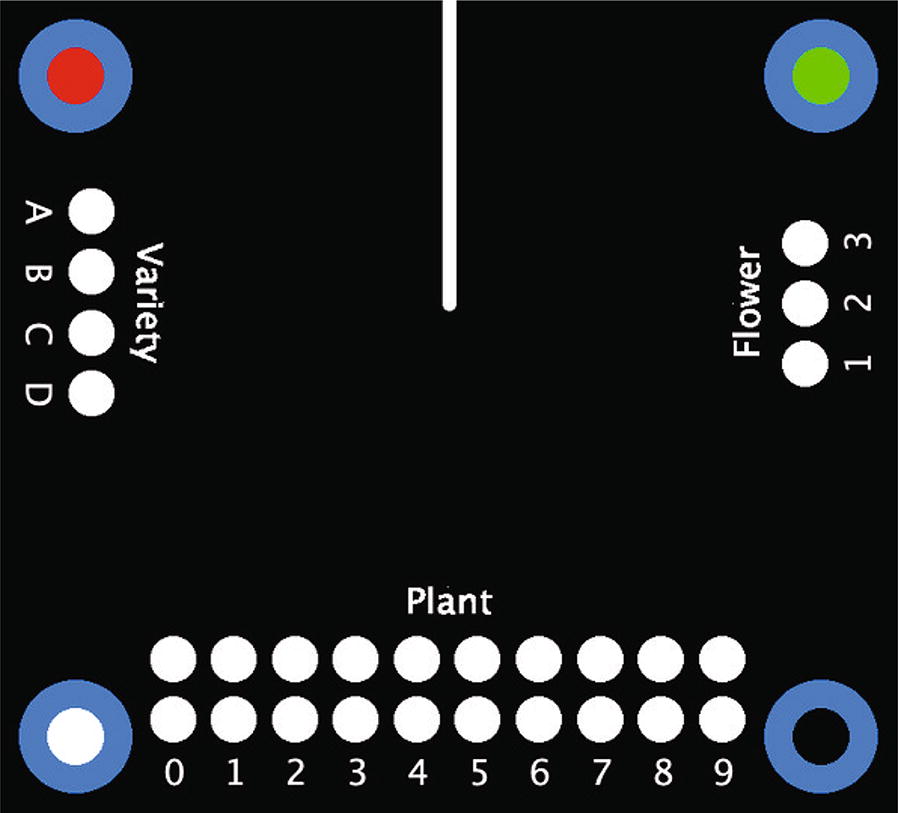
An example SpotCard. This SpotCard contains values in three categories: Variety (values A, B, C, D), Flower (values 1, 2, 3), and Plant (values 0–99, with the top row being ten and the bottom row being units). The line descending from the top of the card is an indicator for where to cut the slit for the plant stem

SpotCards can be created using the SpotCard_Create.ijm macro (Additional file 1). This allows the user to specify the list of categories and values to display on the SpotCard. The macro only asks for three category/value sets at a time, as otherwise the input dialog would become unwieldy. There is no limit within the software to the number of categories and values which can be displayed on the card, although its physical size could become impractical beyond around nine categories or more than around 15 values within a category. (SpotCards are relatively compact: the SpotCard shown in Fig. 1 (set up for a 20 mm diameter flower and contaning two ‘standard’ category/value sets and one ‘integer’ set from 0 to 99) is 62 mm wide × 56 mm high; adding a further three ‘standard’ category/value sets would make it 86 mm wide × 68 mm high).

When creating a SpotCard, the user should first specify the approximate diameter of the flower with which the SpotCard is to be used, with preset options ranging from 20 to 100 mm (though smaller or larger options could be specified by changing the macro code). If the SpotCard is not to be used with specific flowers, this can be set to ‘no flower’.

The user then inputs pairs of categories and values. Categories are simple text-based descriptions of the lists of spots (e.g. ‘Variety’, ‘Plant number’ etc).

There are two ways of specifying values. The first is a simple comma-separated list (e.g. ‘1, 2, 3, 4’ or ‘A, B, C, D’). These will appear on the SpotCard as individual spots, with a value assigned to each. The second way of specifying values is useful when the user wishes to record a large series of integers which it would otherwise be impractical to record with single values (e.g. numbers from 1 to 999). In this case, the value is entered as a range, starting with 0 and ending with a series of 9 s (e.g. 0–999). The macro will output one row of spots for units, one for 10 s, one for 100 s, etc., all within the same category. Integers within the range can then be specified by marking off their individual digits (e.g. to indicate 532, colour the 5 in the 100 s row, the 3 in the 10 s row and the 2 in the units row).

Once categories and values have been specified, the macro creates a TIF image file of the SpotCard. Category names appear above the spots. If the ‘Flower diameter’ value has been set earlier, the sets of spots are arranged around a space in the centre of the SpotCard into which a slit can be cut, so the SpotCard can be slid over the stem of the flower being photographed. Otherwise, the sets are positioned one above the other.

The macro then adds four dots in the corners of the SpotCard, which enable recognition of the SpotCard within the photograph. The default colour for these dots is blue, as it is a colour found rarely in plants, but this can be changed to pink when the SpotCard is to be used to capture information about a blue sample. Each corner dot has a different coloured centre, which allows the processing script to determine the orientation of the SpotCard.

Alongside the TIF file, the macro creates a configuration file for the SpotCard. This file contains information about the X and Y coordinates of the spots, the spot width and height, the diameter of the flower with which the SpotCard is to be used, the spot values, the spot categories, the original width and height of the SpotCard, and the flower centroid Y coordinate. (The flower centroid X coordinate is always half the width of the SpotCard.) This file is read by the second macro and used to locate the positions of the spots and the flower.

**Fig. 2:**
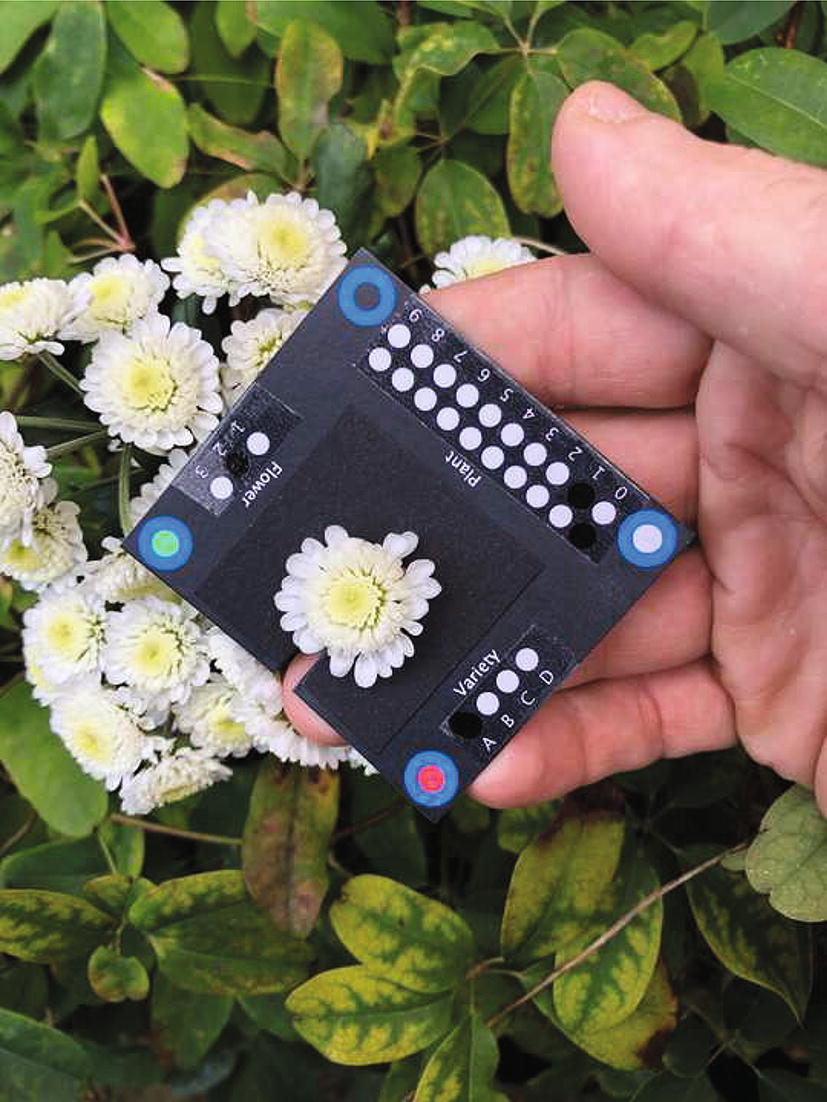
An example image using a SpotCard. The SpotCard can be used at any rotation angle, allowing for easy positioning. Note the tape, to make the spots wipe-clean, and the matt black card behind the flower, reducing reflections which interfere with flower size detection

The user can then print the SpotCard. To make the spots wipe-clean, the card can be laminated; however, reflections from the laminate can interfere with flower trait measurement. Covering the spots with a single layer of sticky tape and affixing matt black card behind the flower area, as in Fig. 2, mitigates this.

### Using the SpotCard

Values are recorded in the spots by colouring them in using a black dry-wipe marker, which requires no solvent to erase. If the card is being used to photograph a flower, the card is then slotted over the stem and the photograph taken. (See Fig. 2 for an example.)

Photographs of plants can include the SpotCard in any orientation. The only constraint is that the SpotCard must be photographed facing directly towards the camera, as the detection macro does not apply any affine transformations to the detected images. However, spot data are still processed correctly even when the SpotCard is tilted up to around 18 degrees around any axis; in practice, it is simple to hold it flatter than this. To facilitate correct processing, corner dots must each be over around 60 px in diameter in the photograph; in practice, if using similar SpotCard positioning to the sample data sets, this equates to images of approximately 1.2 megapixels, easily within the range of modern digital cameras and phones.

### Processing

Once the user has taken photographs, the SpotCard_Analyse.ijm macro (Additional file 2) can be used to detect spot values. This macro uses color thresholding on a brightness- and contrast-adjusted copy of the image to detect the four largest blue areas (which should be the four corner dots). Once the four areas have been located, their centres are examined to detect the colours. The angles of the diagonals of the SpotCards are calculated (i.e. red-centre to black-centre; green-centre to white-centre) and these used to rotate the image so that the corner dot with the red centre is at the top left. The image is then cropped to the blue dots and checked to confirm that the red dot is at the top left of the image. (Thus far, the configuration file has not been used.)

The macro then loops through the positions of all the spots on the image, as specified by the configuration file. For each spot, a selection corresponding to 25% of the spot width and height is examined, which allows for small discrepancies in the positioning of the SpotCard (as it is difficult to take pictures which are exactly face on without using a positioning rig for the camera, flower and SpotCard). The mean gray value is read for a brightness- and contrast-adjusted selection, with a low reading indicating that the spot has been coloured, and a high reading indicating that the spot has been left blank.

Additional flower measurements such as area, perimeter etc. can be performed after the spots have been read. Such analysis can output data in SI units of length and area, rather than simply pixels, as the configuration file gives information which can be used to determine the width and height in millimetres of the SpotCard, thus providing a convenient way of determining the scale of the photograph without including a separate scale bar. The SpotCard_Analyse.ijm code includes area and perimeter recording by way of example, and this has been performed for Sample Data Set 1. Reflections from the printed card may interfere with data detection (as was the case in Sample Data Set 2); affixing matt black card to the area of the SpotCard behind the flower can mitigate this (see Fig. 2). Cropped copies of the flower image and a flood-filled copy of the area used for measurement can be saved for visual confirmation of flower detection accuracy (for examples, see Sample Data Set 1 Flowers and Sample Data Set 1 Flood Fills, available on GitHub at https://github.com/GloverLab/SpotCard).

Once all measuring is complete, the macro saves its results to a text file in CSV format, which can be processed in a variety of other software. Also saved are image filename and, if the EXIF reader plugin (https://imagej.nih.gov/ij/plugins/exif-reader.html) is installed, the date and time the image was taken, and the latitude, longitude and altitude at which the image was taken.

Regular error checking is used throughout the processing script to prevent the reporting of erroneous results. If an error is detected when processing an image, the script will stop processing that image, record the error condition in the output file, and move on to the next image. These checks include:

- Verifying that the lengths of the diagonals between opposing corner dots are within 5% of each other. In practice, this equates to a tilt of 18 degrees around the diagonal of the SpotCard relative to the camera, and it is easy to hold the SpotCard flatter than this.
- Verifying the aspect ratio of the image once cropped to the corner dots matches the configuration file; the recorded aspect ratio and theoretical aspect ratio should differ by no more than 5%. Again, this equates to an 18-degree tilt around the x or y axes of the SpotCard relative to the camera, and it is easy to hold the SpotCard flatter than this.
- Verifying that, once the image has been rotated and cropped, the central portion of the top left dot is red. (This checks that the SpotCard has been correctly oriented.)

## Results

We present two sample sets of results. Processing was performed on a MacBook Pro with a 3.1 GHz Intel Core I7 processor and 8 GB RAM. Sample data sets and the Flowers and Flood Fills images for Sample Data Set 1 are available on GitHub at https://github.com/GloverLab/SpotCard; sample SpotCard images, configuration files and results can be found in Additional files 3, 4, 5, 6, 7, 8).

The first set of results (SampleDataSet1Results.csv) comes from running the detection macro on a set of 75 images of chrysanthemum flowerheads (Sample Data Set 1), taken with an Apple iPhone X. Spots were made wipe-clean by application of clear gloss tape; matt black card was affixed to the area of the SpotCard behind the flower to mitigate reflections causing problems in measurement of flowerhead area and perimeter. The macro took 153 s (1.96 s/image) to correctly process all images with no errors, including flowerhead area and perimeter measurement. Cropped copies of the flowerhead image and a flood-filled copy of the area used for measurement were also saved (Sample Data Set 1 Flowers and Sample Data Set 1 Flood Fills, respectively); visual inspection of these images shows flood-filled areas match the flowerhead images well.

The second set of results (SampleDataSet2Results.csv) comes from running the detection macro on a set of 256 images of strawberry flowers using a fully laminated SpotCard (Sample Data Set 2), taken with an Apple iPhone 8. The macro took 7 min 31 s (1.76 s/image) to correctly read spot data from all except four images, not including flower and perimeter area. Examining these images after processing revealed that one was a badly taken photograph where one corner dot on the SpotCard was obscured by a leaf (Image00094.jpg), one had extraneous blue items in the image (Image00119.jpg), one had reflections on the laminated SpotCard which interfered with the detection (Image00112.jpg), and one had a badly marked spot (Image00151.jpg). All of these four errors could easily be identified from the CSV of results: errors are displayed in the value columns for the first three, and the fourth has a missing value. These images could easily be reprocessed manually or discarded. Flower and perimeter area was not processed for these cards as reflections from the lamination interfered with flower detection.

By comparison, manually reading the filenames and four sets of spot data from all 256 images in Sample Data Set 2 and transcribing them into a spreadsheet took 39 min 40 s (9.3 s/image), five times longer than detection with the macro. There were two transcription errors in the 1024 individual spot entries recorded, a rate of 0.2%. It was impossible to identify these transcription errors by simple inspection of the data, as they were both simple numeric changes (a 1 where there should have been a 2, and a 7 where there should have been an 8). Double-checking would nearly double the time per image and would be necessary to reduce incorrect transcription as a result of human error. The manual processing also ignored flower area and perimeter, date, time and location information; if these were required, manual processing would take further time.

## Conclusion

We have presented SpotCard, a tool which can be used to easily record human- and machine-readable information on a reusable card. Programmatically extracting information from this card can cut the time spent processing such information by at least 80%, and can help eliminate transcription errors caused by human processing. Use cases include analysing flower size of different cultivars of plants, for example in pollinator choice experiments, or of different populations of plants of the same species. It would be interesting to build SpotCard detection into software packages which exist to collect field data, as this could speed up data entry and would enable size measurements of photographed flowers to be made within these software packages.

### Availability and requirements

The tool is open source and available from Github. It requires Fiji [9]. Extraction of date, time and GPS coordinates requires the EXIF reader plugin freely available from https://imagej.nih.gov/ij/plugins/exif-reader.html.

- *Project name*: SpotCard.
- *Project home page*: https://github.com/GloverLab/SpotCard.
- *Operating system(s)*: Any ImageJ/Fiji supports.
- *Programming language*: ImageJ/Fiji macro language.
- *Other requirements*: Developed on Fiji 1.52i.
- *License*: BSD.

## Additional files

**Additional file 1.** The ImageJ macro code to create SpotCards.

**Additional file 2.** The ImageJ macro code to read SpotCards.

**Additional file 3.** The SpotCard used for Sample Data Set 1. (Sample data set available on GitHub).

**Additional file 4.** The configuration file for the SpotCard used in Sample Data Set 1.

**Additional file 5.** The CSV file generated from running SpotCard_Analyse.ijm on Sample Data Set 1. (Sample Data Set 1 Flood Fills and Sample Data Set 1 Flowers are available on GitHub).

**Additional file 6.** The SpotCard used for Sample Data Set 2 (available on GitHub).

**Additional file 7.** The configuration file for the SpotCard used in Sample Data Set 2.

**Additional file 8.** The CSV file generated from running SpotCard_Analyse.ijm on Sample Data Set 2.
