# Supplementary figures and images for: SpotCard: an optical mark recognition tool to improve field data collection speed and accuracy
Source: Plant Methods. 2019 Feb 22;15:19. doi: 10.1186/s13007-019-0403-2 (PMC6385457; doi:10.1186/s13007-019-0403-2)

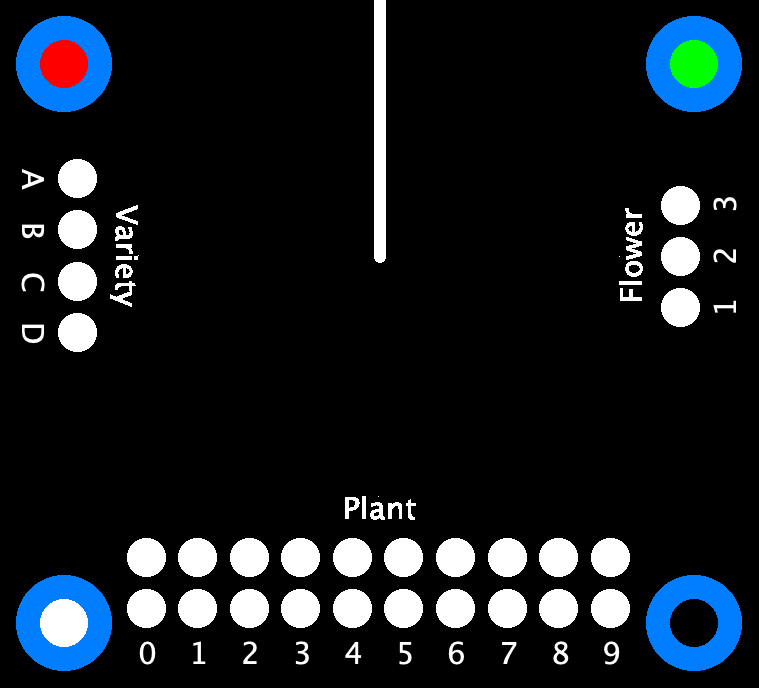

Supplement: Supplementary file 3 — Additional file 3. The SpotCard used for Sample Data Set 1. (Sample data set available on GitHub). [file 13007_2019_403_MOESM3_ESM.tif]

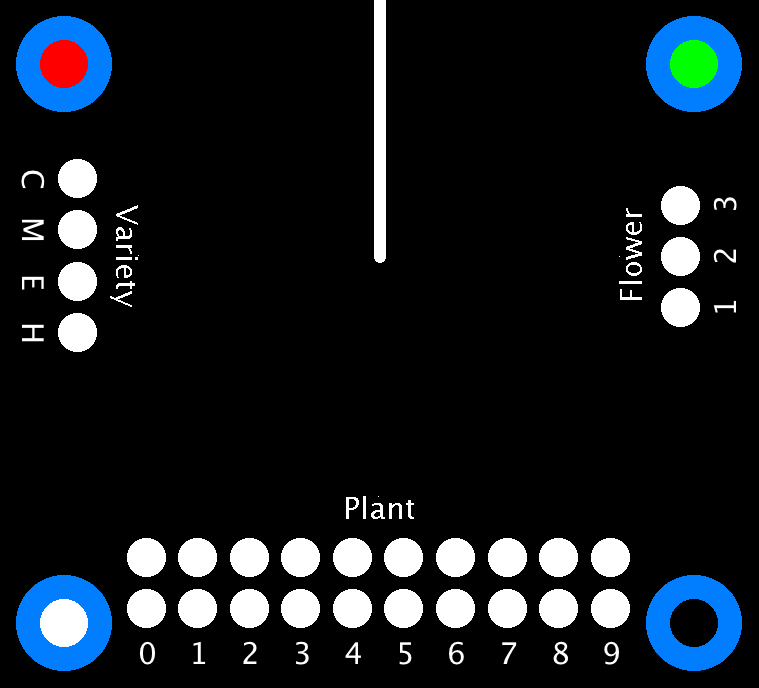

Supplement: Supplementary file 6 — Additional file 6. The SpotCard used for Sample Data Set 2 (available on GitHub). [file 13007_2019_403_MOESM6_ESM.tif]
